# Automated quantitative pupillometry as a predictor for transtentorial brain herniation in patients with malignant acute ischemic stroke

**DOI:** 10.1371/journal.pone.0316358

**Published:** 2025-01-10

**Authors:** Catherine Park, So Young Park, Min Kim, Bumhee Park, Ji Man Hong

**Affiliations:** 1 Department of Convergence of Healthcare and Medicine, Ajou University Graduate School of Medicine, Suwon, South Korea; 2 Division of Digital Healthcare, Yonsei University, Wonju, South Korea; 3 Department of Neurology, Ajou University School of Medicine, Suwon, South Korea; 4 Department of Biomedical Informatics, Ajou University School of Medicine, Suwon, South Korea; Aga Khan University Hospital, PAKISTAN

## Abstract

Brain herniation can be a life-threatening condition, resulting in poor prognosis and higher fatality rates. We examined whether quantitative characteristics of sequential pupillary light reflex (PLR) could serve as biomarkers for identifying brain herniation in fatal acute stroke cases with anterior circulation involvement admitted to neurological intensive care unit (Neuro-ICU). Automatic pupillometer assessed PLR automatically every 4–6 hours, measuring eight specific features: NPi (Neurological pupil index) score, initial resting and constriction pupil size, constriction change, constriction velocity, constriction latency, and dilation velocity. Generalized estimating equations were used to analyze the main effects of assessment time (3-to-0 hours, just before brain herniation, and 27-to-21 hours, considerably before) and clinical groups. The study involved 59 patients (mean age 68.8 ± 1.6 years, 23 females) divided into herniation (n = 10) and non-herniation (n = 49) groups. The herniation group exhibited significantly lower ipsilateral NPi scores at 3-to-0 hours (1.80 ± 0.44, p < 0.0001) compared to 27-to-21 hours (4.26 ± 2.21). Additionally, the herniation group had a larger ipsilateral pupil size at constriction at 3-to-0 hours (4.01 ± 0.40 mm) compared to 27-to-21 hours (2.11 ± 0.17 mm). Specifically, at 3-to-0 hours, the herniation group had lower NPi scores (1.80 ± 0.44 vs. 3.97 ± 0.13, p < 0.0001) and larger pupil size at constriction (4.01 ± 0.04 mm vs. 2.90 ± 0.10 mm, p = 0.007) compared to the non-herniation group. These findings suggest that evaluating PLR characteristics can aid in the early identification of brain herniation, facilitating timely triage and appropriate surgical management.

## 1. Introduction

Brain herniation is a serious medical condition where brain tissue is displaced from its normal position due to increased intracranial pressure (ICP) [1, 2]. This can happen in stroke patients when there is significant cerebral edema (swelling) or large space-occupying lesions in the brain [2, 3]. Brain herniation is a life-threatening condition and can cause various neurological manifestations (e.g., alterations in consciousness, abnormal pupillary responses, cranial nerve abnormalities, motor deficits, etc.) [4], leading to a poor prognosis and increased mortality rate [5–11]. Thus, timely detection of brain herniation and immediate medical intervention are crucial in improving the chances of survival in acute stroke patients admitted to the neurological intensive care unit (Neuro-ICU).

The diagnosis of brain herniation typically involves a comprehensive approach, utilizing various diagnostic modalities, such as advanced brain imaging techniques like CT and MRI scans [12, 13]. Additionally, medical doctors closely monitor ICP as an essential indicator for detecting brain herniation [14, 15]. Moreover, clinical assessments play a crucial role in identifying brain herniation, involving the evaluation of the patient’s level of consciousness, pupillary reactions (i.e., Pupillary Light Responses (PLR)), eye movements, and motor and sensory responses [1, 16, 17]. These diagnostic methods are effective but may face delays due to resource constraints. Logistical challenges, such as mobility constraints and resource demands (e.g., time, cost, labor, etc.), can hinder the prompt implementation of brain imaging and ICP monitoring for accurate diagnosis and timely management of brain herniation cases.

Automated pupillometry, also known as an automated PLR device, has several advantages for early brain herniation detection, including objective and quantitative measurements, non-invasive and rapid assessment, and cost-effectiveness. Previous studies have shown its efficacy in identifying neurological conditions like brain herniation, intracranial hypertension, aneurysmal subarachnoid hemorrhage, and spontaneous intracerebral hemorrhage [11, 18–25]. However, little is known about the specific and sequential PLR characteristics in patients experiencing brain herniation over time. Additionally, the use of automated pupillometry in the Neuro-ICU for identifying brain herniation may be limited by false negatives, confounding variables, and a lack of predictive value [21, 26].

Recognizing the aforementioned limitations, the importance of analyzing both ipsilateral, and contralateral PLR [1, 27] and the need for continuous monitoring over time in the Neuro-ICU for early brain herniation identification [28–30], this study aimed to investigate the quantitative characteristics of PLR as a biomarker for identifying and predicting brain herniation in acute stroke patients with malignant infarct core. Specifically, the study measured different variables related to PLR on both sides over time, explored the association between these characteristics and brain herniation identification, and examined statistical models for predicting brain herniation.

## 2. Methods

### 2.1. Participants

This is a retrospective study from automated pupillary response on Neuro-ICU (APRoN) registry of Ajou University Hospital. The study included acute malignant ischemic stroke admitted to the Neuro-ICU, a tertiary referral stroke center between December, 2018 and May, 2023. The study was conducted according to the guidelines of the Declaration of Helsinki and approved by the Institutional Review Board of Ajou University Hospital. Since this retrospective study used anonymized data, written informed consent for participation was not required for this study with the national legislation and the institutional requirements.

Participants were males and females of all racial and ethnic backgrounds who met specific criteria; (1) having acute ischemic stroke involving malignant at least >½ middle cerebral artery territory on initial CT; (2) having diffusion weighted image volume > 82 mL within 6 hours after neurological symptom onset (i.e., hemiplegia, aphasia, and/or mental change) [31, 32]; (3) having middle cerebral artery occlusion in anterior circulation involvement; and/or (4) having internal carotid artery occlusion. Participants also underwent at least 6 PLR examinations using an automated pupillometer for the past 48 hours before the CT scan for brain herniation diagnosis.

Potential participants were excluded if they had status epilepticus, encephalitis, or a neuromuscular disorder. Daily brain CT scans were conducted according to the Ajou Stroke Team Protocol for all patients, except those deemed clinically unsuitable due to unstable vital signs or undergoing targeted temperature management. Furthermore, if anisocoria or the presence of a sluggish pupil was confirmed in the sequential pupillometry measurements, a brain CT scan was conducted within 3 hrs. Brain herniation was radiologically defined as a case where the medial temporal lobe or occipital lobe horizontally compressed the brain stem, including the midbrain, corresponding to transtentorial uncal herniation in brain CT scans within 24 to 48 hours post-stroke onset [33]. This retrospective study utilized Electronic Health Record (EHR) and was approved by the Institutional Review Board of Ajou University Medical Center.

Demographic information (age, sex, and body mass index), clinical characteristics (affected brain side, treatments received modified Rankin Scale (mRS) for outcome measure, preventive medication, risk factors (hypertension, diabetes mellitus, dyslipidemia, smoking status, atrial fibrillation, heart conditions), and laboratory factors (lipid profiles, hemoglobin A1C, prothrombin time, etc.) were collected for analysis.

### 2.2. Pupillary variables

**Fig 1:**
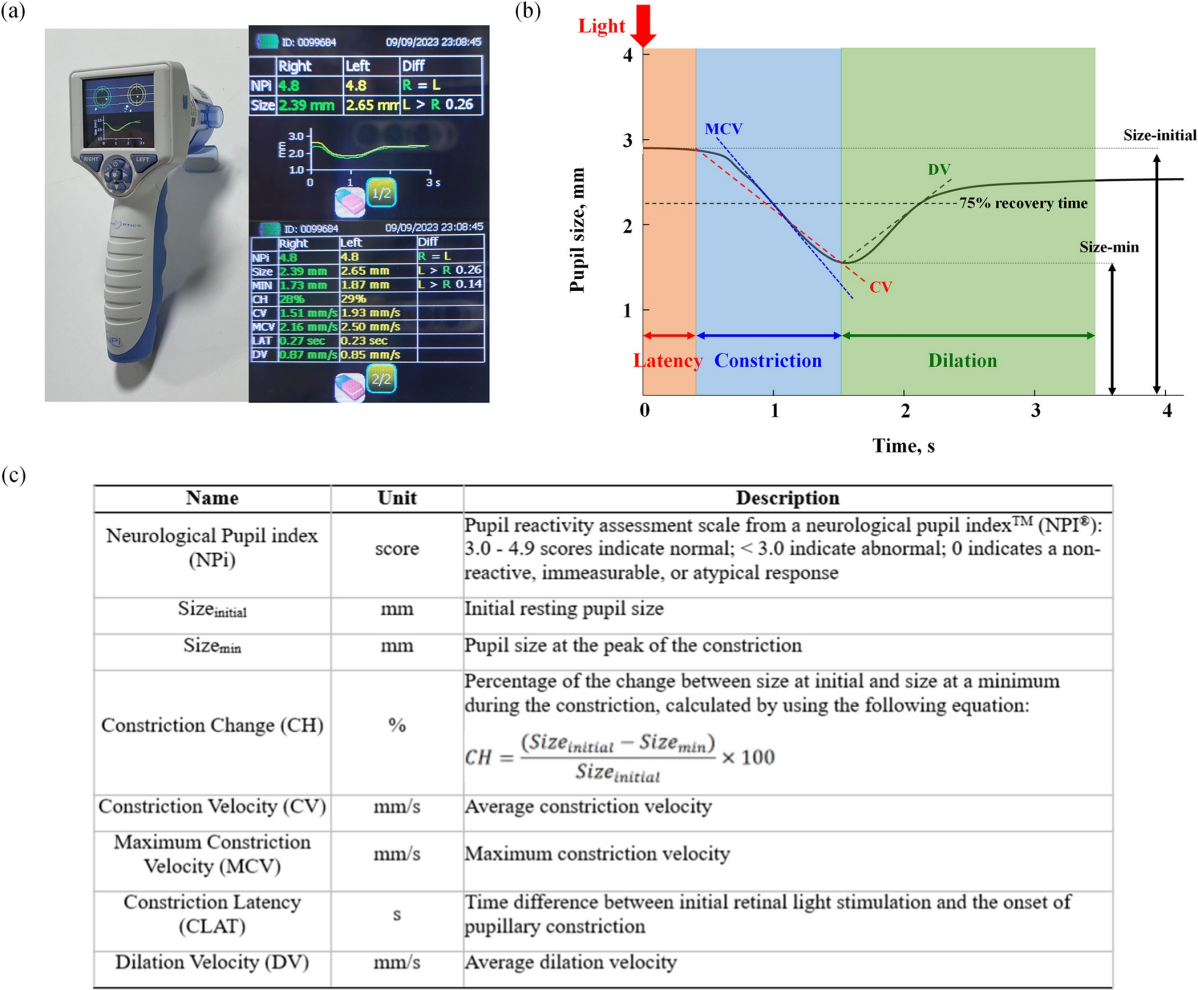
(a) Automated pupillometer (NeurOptics NPi®-200, Neuroptics Inc., Irvine, USA) for pupil reactivity assessment and an example of the measured values. (b) Pupil size and reactivity over time. (c) Description of eight pupillary measurement variables.

**Fig 1** presents the automated pupillometer (NeurOptics NPi®-200, Neuroptics Inc., Irvine, USA) for pupil reactivity assessment and an example of the measured values, pupil size and reactivity over time, and description of eight pupillary measurement variables [34, 35]. These variables include the Neurological Pupil index (NPi) scores categorized as normal (3.0–4.9), abnormal (< 3.0), and non-reactive, immeasurable, or atypical response (0). Additionally, we measured pupil size at initial resting (Size-initial), pupil size at constriction (Size-min), constriction change (CH), constriction velocity (CV), maximum constriction velocity (MCV), constriction latency (CLAT), and dilation velocity (DV).

The measurements were conducted on both the ipsilateral and contralateral sides of the stroke-affected brain at specific time intervals before CT scan for brain herniation diagnosis. The timeframes for measurements were 48-to-33, 33-to-27, 27-to-21, 21-to-15, 15-to-9, 9-to-3, and 3-to-0 hours before the identification of the brain herniation. We defined 0-hour as the time that the clinician diagnosed herniation based on the results of the CT scan for the herniation group and the time that the clinician conducted the last PLR test for the non-herniation group. Due to practical circumstances in the Neuro-ICU, patients with brain herniation showed sudden collapsed pupillary light reflexes a few hours before the identification of the brain herniation using a CT scan, and clinicians needed to triage patients with at-risk herniation. Thus, we chose 3-to-0 hours (i.e., 3-hour duration) before the identification of the brain herniation. Note that uniformly distributed timeframes such as 36-to-30, 30-to-24, 24-to-18, 18-to-12, 12-to-6, and 6-to-0 hours before the identification of the brain herniation were also analyzed (**S1 Fig** and **S1 Table**). Participants were categorized into two groups such as herniation and non-herniation based on the CT scan results.

### 2.3. Data and statistical analysis

MATLAB (MathWorks, Natick, MA, USA) and IBM SPSS Statistics (IBM Corp., Armonk, NY, USA) were used to conduct data and statistical analysis. Outcome measures were the participants’ demographics and the eight pupillary variables computed over seven measurement timeframes (48-to-33, 33-to-27, 27-to-21, 21-to-15, 15-to-9, 9-to-3, and 3-to-0 hours).

The Shapiro-Wilk test was used to identify the normal distribution of continuous variables. To determine differences of demographics, clinical characteristics, risk factors, and laboratory factors between groups (i.e., herniation and non-herniation groups), a one-way analysis of variance (ANOVA) for normally distributed variables or a Mann-Whitney U test for non-normally distributed variables was conducted. A chi-square test was performed to test categorical variables for significant levels between groups. Generalized estimating equations (GEE) were used to assess the main effects of the measurement timeframe (i.e., 48-to-33, 33-to-27, 27-to-21, 21-to-15, 15-to-9, 9-to-3, and 3-to-0 hours) and the group (herniation and non-herniation groups) as well as their interactions. The generalized estimating equations allow for testing differences between groups as well as at specific time points for the longitudinal design and are valid for missing data at a particular patient or time at random [36]. To assess accelerated deterioration in PLR, we compared the eight pupillary variables for the seven measurement timeframes for both groups. For this analysis, multiple pairwise comparisons using the least significant difference method were conducted. Binary logistic regression modeling with a forward selection method was conducted to examine the association between the eight pupillary variables and the identification of brain herniation. Four binary logistic regression models were designed to identify and predict brain herniation. In model 1, the pupil size at initial resting (Size-initial) data at the 27-to-21 hour timeframe were used as the independent variable. In model 2, the dilation velocity (DV) data at the 15-to-9 hour timeframe were used as the independent variable. In model 3, the dilation velocity (DV) data at the 9-to-3 hour timeframe were used as the independent variable. In model 4, the constriction change (CH) or NPi score data at the 3-to-0 hour timeframe was used as the independent variable. It is worth noting that the forward selection method determined the best pupillary variable for accurately identifying and predicting brain herniation at each timeframe. Thus, Size-initial, DV, DV, and CH were used for models 1, 2, 3, and 4, respectively. Additionally, the NPi score was included in the model 4, because it is a direct outcome of the automated pupillometer (NeurOptics NPi®-200, Neuroptics Inc., Irvine, USA). To evaluate the performance of the four binary logistic regression models, the receiver operating characteristic (ROC) curve and area under the curve (AUC) were calculated. An AUC value between 0.8 and 0.9 was considered excellent, while an AUC value higher than 0.9 was considered outstanding [37]. The significance level was set at 2-sided p < 0.05 for all statistical analyses.

## 3. Results

### 3.1. Demographic and clinical characteristics

**Fig 2:**
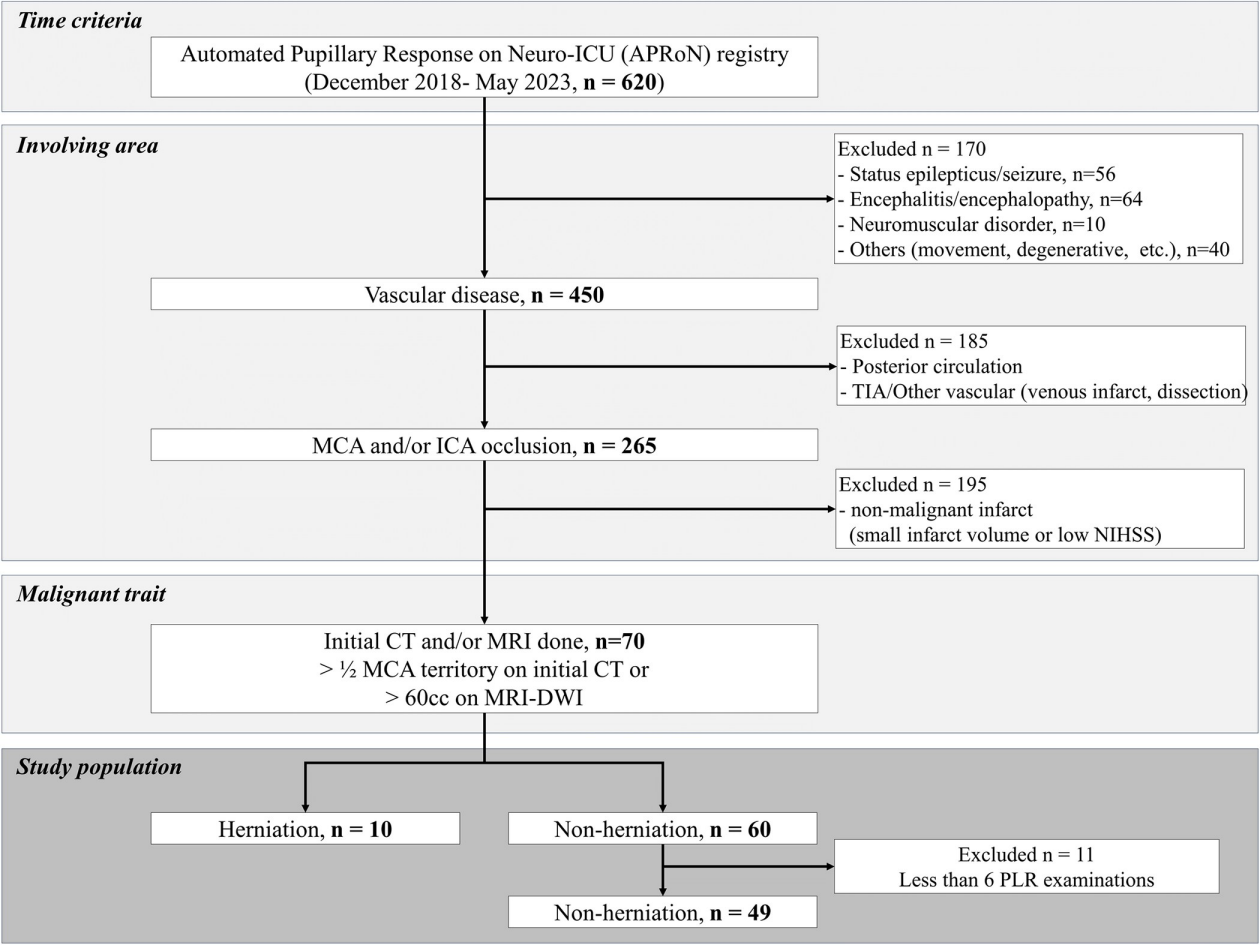
Strobe diagram of patient eligibility determinations for analysis.

Of 620 patients admitted to the Neuro-ICU, 70 met the inclusion criteria. Among them, 10 participants were diagnosed with brain herniation (herniation group), while 49 participants did not have brain herniation (non-herniation group) (**Fig 2**). **Table 1** presents the demographics and clinical characteristics of both groups, along with the corresponding statistical results. The analysis revealed that the discharge mRS was significantly higher in the herniation group compared to the non-herniation group. However, there were no significant differences between the groups in terms of demographics, other clinical characteristics (excluding discharge mRS), risk factors, and laboratory factors.

**Table 1:** Demographics, clinical characteristics, risk factors, and laboratory factors for the herniation and non-herniation groups.

	Patients, No./Total No. (%), by group			
	Herniation group (n = 10)	Non-Herniation group (n = 49)	*p* value	*OR* *(95% CI)*
**Demographics**				
Age, *years*	70.1 ± 4.2	68.5 ± 1.7	0.700	-
Sex (Female), n (%)	3 (30.0)	20 (40.8)	0.523	0.621 (0.143–2.696)
BMI (kg/m^2^)	24.0 ± 2.3	22.6 ± 0.5	0.331	-
**Clinical characteristics**				
Lesion location, n (%)				
Isolated MCA	6 (60.0)	44 (89.8)	0.036	-
ICA involvement	7 (70.0)	24 (49.0)	0.306	-
Affected side (Left), n (%)	3 (30.0)	19 (38.8)	0.601	0.677 (0.156–2.942)
Infarct volume, mL	255.7 ± 89.1	165.9 ± 86.9	0.012	-
Initial NIHSS (IQR)	22 (19, 22)	19 (16, 21)	0.157	-
Administration of tPA, n (%)	3 (30.0)	-	-	-
Intra-arterial therapy (IAT), n (%)	7 (70.0)	-	-	-
Recanalization, n (%)	3 (30.0)	-	-	-
Active TTM, n (%)	6 (60.0)	-	-	-
Therapeutic drugs				
Midazolam	10 (100)	38 (77.6)	0.183	
Propofol	5 (50)	30 (61.2)	0.726	
Opioid	3 (30)	30 (61.2)	0.090	
Osmolar therapy	10 (100.0)	21 (42.9)	<0.001	
Suspected herniation to CT, *minutes*	118.1 ± 85.9			
Onset to herniation, *days*	2 (2, 3)	-	-	-
Decompressive craniectomy	2 (20.0)	1 (2.0)	0.072	
Premorbid mRS	0.22 ± 0.22	0.71 ± 0.17	0.181	-
Discharge mRS	5.44 ± 0.29	4.57 ± 0.12	0.003	-
**Stroke risk factors**				
Hypertension, n (%)	5 (50.0)	32 (65.3)	0.362	0.531 (0.135–2.095)
Diabetes mellitus, n (%)	2 (20.0)	14 (28.6)	0.578	0.625 (0.118–3.316)
Dyslipidemia, n (%)	3 (30.0)	18 (36.7)	0.685	0.738 (0.169–3.216)
Atrial Fibrillation, n (%)	4 (40.0)	28 (57.1)	0.321	0.500 (0.125–1.999)
CAD, n (%)	1 (10.0)	5 (10.2)	0.984	0.978 (0.102–9.404)
Previous stroke or TIA, n (%)	1 (10.0)	10 (20.4)	0.441	0.433 (0.049–3.832)
**Laboratory factors**				
HbA1c	5.8 ± 0.7	6.1 ± 1.1	0.607	-
Total cholesterol	150.0 ± 39.7	167.2 ± 36.9	0.211	-
Triglyceride	109.8 ± 49.7	124.6 ± 92.9	0.838	-
High-density lipoprotein	49.7 ± 7.8	48.7 ± 16.6	0.862	-
Low-density lipoprotein	78.4 ± 30.9	93.8 ± 33.0	0.203	-

Values are presented as mean ± standard error or n (%). BMI: body mass index, MCA: Middle cerebral artery, ICA: Internal carotid artery, NIHSS: National institutes of health stroke scale, IQR: interquatile range, tPA: tissue plasmin activator, TTM: Targeted temperature management, mRS: Modified Rankin Scale, CAD: Coronary artery disease, TIA: Transient ischemic attack, HbA1c: Hemoglobin A1c.

### 3.2. Within- and between-group comparison of pupillary variables

**Table 2** presents the primary results of the statistical analysis for the eight pupillary variables, including the main effects of the group and measurement timeframe as well as the interaction effects (group × measurement timeframe). The analysis included pairwise comparisons between the herniation and non-herniation groups at two specific timeframes (i.e., 27-to-21 and 3-to-0 hours).

**Table 2:** Statistical analysis results of eight pupillary variables as a function of group (G), measurement timeframe (T), and their interaction (G × T) between different timeframes (i.e., 27-to-21 hour vs. 3-to-0 hour) for the ipsilateral and contralateral sides. Values are presented as mean ± standard error. An asterisk denotes statistical significance.

	Main and Interaction	Herniation group (*n* = 10)			Non-Herniation group (*n* = 49)			Herniation group vs. Non-Herniation group	
	Group (G)—Main Measurement timeframe (T)—Main G × T—Interaction	Considerably-before herniation: 27-to-21 hour	Just-before herniation: 3-to-0 hour	*p* value	Considerably-before herniation: 27-to-21 hour	Just-before herniation: 3-to-0 hour	*p* value	Considerably-before herniation: 27-to-21 hour *p* value	Just-before herniation: 3-to-0 hour *p* value
**NPi**									
Ipsilateral	G = 0.236 T < 0.0001* G × T < 0.0001*	4.26 ± 2.21	1.80 ± 0.44	< 0.0001*	4.01 ± 0.13	3.97 ± 0.13	0.718	0.301	< 0.0001*
Contralateral	G = 0.915 T = 0.009* G × T = 0.002*	4.37 ± 0.13	3.47 ± 0.32	0.004*	4.01 ± 0.14	3.96 ± 0.12	0.686	0.050	0.152
**Size-initial**									
Ipsilateral	G < 0.0001* T = 0.001* G × T = 0.001*	2.61 ± 0.18	4.22 ± 0.41	< 0.0001*	4.00 ± 0.14	4.04 ± 0.12	0.742	< 0.0001 *	0.671
Contralateral	G < 0.0001* T = 0.013* G × T **=** 0.001*	2.51 ± 0.26	2.95 ± 0.49	0.433	4.06 ± 0.15	4.02 ± 0.13	0.772	< 0.0001 *	0.036 *
**Size-min**									
Ipsilateral	G = 0.185 T < 0.0001* G × T < 0.0001*	2.11 ± 0.17	4.01 ± 0.40	< 0.0001*	2.85 ± 0.11	2.90 ± 0.10	0.588	< 0.0001*	0.007*
Contralateral	G < 0.0001* T = 0.038* G × T = 0.020*	1.98 ± 0.17	2.54 ± 0.34	0.109	2.86 ± 0.11	2.89 ± 0.10	0.739	< 0.0001*	0.318
**CH**									
Ipsilateral	G < 0.0001* T < 0.0001* G × T < 0.0001*	18.57 ± 2.43	5.31 ± 0.82	< 0.0001*	28.23 ± 1.42	28.04 ± 1.38	0.878	0.001*	< 0.0001*
Contralateral	G < 0.0001* T = 0.009* G × T = 0.013*	19.33 ± 2.83	11.28 ± 3.67	0.123	28.87 ± 1.42	27.56 ± 1.30	0.267	0.003*	< 0.0001*
**CV**									
Ipsilateral	G < 0.0001* T < 0.0001* G × T < 0.0001*	1.01 ± 0.14	0.35 ± 0.08	< 0.0001*	2.07 ± 0.12	2.06 ± 0.13	0.938	< 0.0001*	< 0.0001*
Contralateral	G < 0.0001* T = 0.003* G × T **=** 0.001*	1.11 ± 0.18	0.69 ± 0.28	0.237	2.21 ± 0.14	2.07 ± 0.13	0.190	< 0.0001*	< 0.0001*
**MCV**									
Ipsilateral	G < 0.0001* T < 0.0001* G × T < 0.0001*	1.61 ± 0.22	0.75 ± 0.07	< 0.0001*	3.08 ± 0.18	3.35 ± 0.21	0.110	< 0.0001*	< 0.0001*
Contralateral	G < 0.0001* T = 0.001* G × T < 0.0001*	1.59 ± 0.25	1.06 ± 0.40	0.315	3.28 ± 0.19	3.15 ± 0.18	0.425	< 0.0001*	< 0.0001*
**CLAT**									
Ipsilateral	G = 0.142 T = 0.065 G × T **=** 0.148	0.28 ± 0.02	0.38 ± 0.05	0.052	0.25 ± 0.01	0.27 ± 0.01	0.150	0.144	0.039*
Contralateral	G = 0.024* T = 0.948 G × T = 0.415	0.29 ± 0.02	0.30 ± 0.02	0.924	0.26 ± 0.01	0.27 ± 0.01	0.584	0.082	0.260
**DV**									
Ipsilateral	G = < 0.0001* T < 0.0001* G × T < 0.0001*	0.49 ± 0.08	0.18 ± 0.03	< 0.0001*	0.86 ± 0.05	0.96 ± 0.05	0.032 *	< 0.0001*	< 0.0001*
Contralateral	G < 0.0001* T = 0.006* G × T < 0.0001*	0.47 ± 0.09	0.29 ± 0.12	0.272	0.88 ± 0.05	0.96 ± 0.06	0.087	< 0.0001*	< 0.0001*

The results of the statistical analysis indicated the main effects of the group for ipsilateral Size-initial, CH, CV, MCV, and DV and for contralateral Size-initial, Size-min, CH, CV, MCV, LAT, and DV. They also indicated the main effects of the measurement timeframe for ipsilateral and contralateral NPi score, Size-initial, Size-min, CH, CV, MCV, and DV. Furthermore, the results indicated interaction effects for ipsilateral and contralateral NPi score, Size-initial, Size-min, CH, CV, MCV, and DV.

The results of pairwise comparisons between the two timeframes (i.e., 27-to-21 and 3-to-0 hours) for each group indicated that the herniation group had significantly lower ipsilateral NPi score, CH, CV, MCV, and DV, higher ipsilateral Size-initial and Size-min, and lower contralateral CLAT at the 3-to-0 hour timeframe compared to the 27-to-21 hour timeframe. The results also indicated that the non-herniation group had a significantly higher value for only DV at the 3-to-0 hour timeframe compared to the 27-to-21 hour timeframe.

The results of pairwise comparisons between groups as a function of the two measurement timeframes (i.e., 27-to-21 and 3-to-0 hours) indicated that the herniation group had significantly lower ipsilateral and contralateral Size-initial, Size-min, CH, CV, MCV, and DV than the non-herniation group at the 27-to-21 hour timeframe. At the 3-to-0 hour timeframe, the herniation group had significantly lower ipsilateral NPi score, CH, CV, MCV, and DV, higher ipsilateral Size-min and CLAT, and lower contralateral Size-initial, CH, CV, MCV, and DV than the non-herniation group.

### 3.3. Pupil responses on the ipsilateral and contralateral sides as a function of time

**Fig 3:**
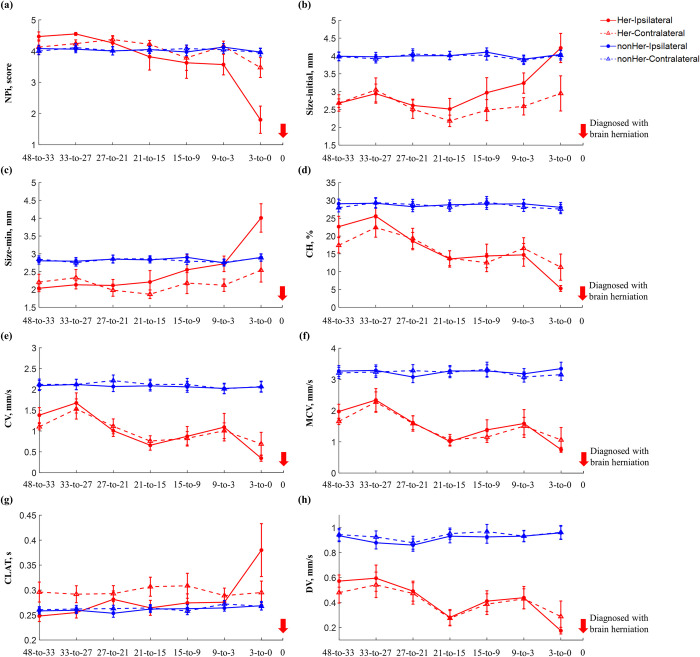
Comparing features for pupil response on the ipsilateral (stroke-affected) and contralateral sides across the past 48 hours between herniation (Her, red color) and non-herniation groups (nonHer, blue color). (a) NPi; (b) Size-initial; (c) Size-min; (d) CH; (e) CV; (f) MCV; (g) CLAT; and (h) DV. Error bars indicate the standard error of the corresponding mean.

**Fig 3** shows eight pupillary variables on the ipsilateral and contralateral sides for herniation and non-herniation groups over seven measurement timeframes. **S2 Table** presents pairwise comparisons of the eight pupillary variables on the ipsilateral and contralateral sides in the herniation group. **S3 Table** presents pairwise comparisons of the eight pupillary variables on the ipsilateral and contralateral sides in the non-herniation group.

The closer the herniation was detected, the lower NPi score, CH, CV, MCV, and DV and the higher Size-initial, Size-min, and CLAT on the ipsilateral side. However, the eight pupillary variables on the contralateral side were not significantly different between the measurement timeframes.

### 3.4. Performance of models for predicting brain herniation

**Fig 4:**
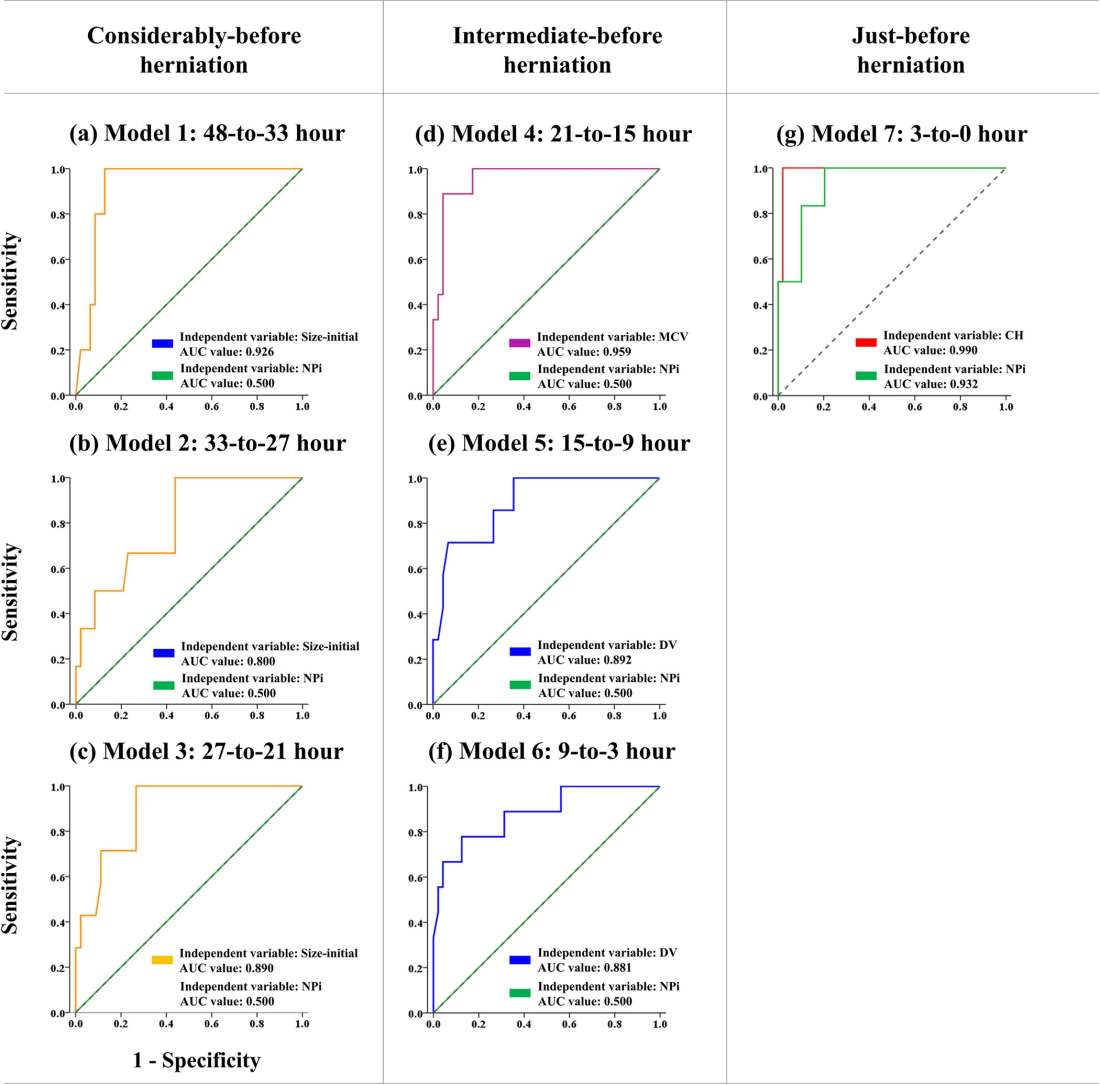
Performance of seven binary logistic regression models with ipsilateral PLR variables, including an AUC value. (a) Model 1: Size-initial at 48-to-33 hour timeframe; (b) Model 2: Size-initial at 33-to-27 hour timeframe; (c) Model 3: Size-initial at the 27-to-21 hour timeframe; (d) Model 4: MCV at 21-to-15 hour timeframe; (e) Model 5: DV at the 15-to-9 hour timeframe; (f) Model 6: DV at the 9-to-3 hour timeframe; (g) Model 7: CH at the 3-to-0 hour timeframe. All models include NPi for comparison.

**Fig 4** shows the results of the performance analysis of seven statistical models with ipsilateral pupillary variables. The models with Size-initial at 48-to-33 hour timeframe, Size-initial at 33-to-27 hour timeframe, Size-initial at the 27-to-21 hour timeframe, MCV at 21-to-15 hour timeframe, DV at the 15-to-9 hour timeframe, and DV at the 9-to-3 hour timeframe achieved excellent AUC values of 0.93, 0.80, 0.89, 0.96, 0.89, and 0.88, respectively (**Fig 4A–4F**).

Notably, the models with CH at the 3-to-0 hour timeframe resulted in outstanding AUC for predicting brain herniation (**Fig 4G**). The AUC for the CH model surpassed that of the NPi score model indicating superior predictive performance (i.e., 0.99 vs. 0.93, as shown in **Fig 4G**).

## 4. Discussion

This study explored pupillary variable changes in acute ischemic stroke patients with malignant core, anticipating brain herniation. The herniation group showed significant changes in NPi score, Size-initial, Size-min, CH, CV, MCV, and DV on the ipsilateral side, comparing 3-to-0 hours (just-before) to 27-to-21 hours (considerably-before) before brain herniation identification. Ipsilateral pupillary responses may be more sensitive indicators of brain herniation. Notably, significant differences in pupillary variables at the 27-to-21 hour suggest early indicators for timely interventions. At the 3-to-0 hour, additional pupillary variables differed significantly between herniation and non-herniation groups, emphasizing their importance in monitoring brain herniation progression. Statistical models with CH or NPi score at the 3-to-0 hour demonstrated outstanding performance in predicting brain herniation (i.e., 0.99 vs. 0.93), with CH outperforming NPi score. The study highlighted the critical nature of brain herniation, with shorter survival time in the herniation group, underscoring the need for early detection and interventions. Pupillary variables hold potential as predictive indicators for acute malignant ischemic stroke patients at risk of brain herniation, aiding informed decision-making by healthcare providers.

In general, the onset and timing of brain herniation in stroke patients may vary significantly depending on several factors, including the size and location of the stroke, the presence of underlying conditions, the effectiveness of medical interventions, and individual patient characteristics. However, previous studies have reported that large-scale cerebral infarction in malignant ischemic stroke patients can lead to brain herniation within a few days after its onset [38–40]. Therefore, continuous monitoring can provide insights into the development of brain herniation in acute malignant ischemic stroke patients and allow healthcare providers to implement timely medical interventions (e.g., pharmacological treatments, therapeutic hyperventilation, or even surgical interventions) aimed at reducing ICP and preventing herniation. This study revealed that the herniation group showed a significant change in pupillary variables except for CLAT from the 27-to-21 hour to the 3-to-0 hour, which suggests that symptoms may start to manifest approximately one day before a brain herniation is identified. In addition, the pupillary variables used in this study showed a significant difference between the groups at the 27-to-21 hour and 3-to-0 timeframes, indicating that the pupillary variables could be reliable to identify the potential onset of brain herniation.

During brain herniation, ICP can compress the oculomotor nerve at the level of the midbrain. This compression can lead to dysfunction or paralysis of the parasympathetic fibers that innervate the constrictor muscles of the iris, causing dilation of the affected pupil (i.e., ipsilateral pupillary dilation). One retrospective study found an association between ipsilateral NPi abnormalities and malignant cerebral edema [41]. Another study also found that abnormal NPi scores were associated with acute cerebral edema [42]. Additionally, studies revealed that a lower NPi score and a sudden decrease in the NPi were predictors of neurological deterioration during follow-up [1, 30, 43, 44]. Moreover, multiple studies have indicated that NPi scores may be utilized to identify or predict neurological outcomes (e.g., brain herniation, intracranial hypertension, aneurysmal subarachnoid hemorrhage, and spontaneous intracerebral hemorrhage) [1, 18–21, 45]. However, these studies have only relied on NPi scores. Compared to these studies, therefore, this study revealed significant changes in ipsilateral NPi score, Size-initial, Size-min, CH, CV, MCV, and DV between the 27-to-21 hour and the 3-to-0 hour in the herniation group. Although the contralateral pupil may also be affected by brain herniation, the results of this study showed that only the NPi score was significantly lower in the herniation group between the 27-to-21 hour and the 3-to-0 hour. Taken together, the results of this study suggest that ipsilateral Size-initial, Size-min, CH, CV, MCV, and DV need to be examined in conjunction with the NPi score. Furthermore, the results of statistical models with ipsilateral pupillary variables suggest that pupillary size (i.e., Size-initial) and velocity (i.e., MCV and DV) could be a potential biomarker to identify the risk of brain herniation at the considerably before herniation (i.e., 48-to-33, 33-to-27, and 27-to-21) and the intermediate before herniation (i.e., 21-to-15, 15-to-9, and 9-to-3), respectively. Just before herniation, the change between size at initial and size at a minimum during the constriction (i.e., CH) could be a potential biomarker.

A recent review has documented the potential use of pupillometry as a prognostic indicator, particularly in cases of cardiac arrest and acute brain injuries, including brain herniation [18]. This review concluded that the NPi offers an objective and automated evaluation of PLR, which can overcome the subjectivity and technician-dependent nature of conventional methods of pupil assessment. Yet, exploring statistical models and assessing other pupillary parameters (e.g., pupil size, constriction change, contraction velocity, dilation velocity) for predicting brain herniation has not been studied. The performance results of models for predicting brain herniation indicated that evaluating Size-initial at the 27-to-21 hour and DV at the 27-to-21 hour could potentially predict the incidence of brain herniation in acute malignant ischemic stroke patients. At the 3-to-0 hour, CH was identified to best predict brain herniation. Notably, the performance of the model with CH was superior to the performance of the model with the NPi score. These results suggest that other pupillary parameters (i.e., Size-initial, DV, and CH) in conjunction with the NPi score could be effective potential biomarkers to predict brain herniation.

The limitations of this study are a relatively small sample size for the herniation group and patient cohort (i.e., acute malignant ischemic stroke patients) and the unequal sample size between groups. The examination time of PLR using an automated pupillometer within each timeframe was not consistent across the patients. Furthermore, it is essential to exercise caution when interpreting the results, taking into account potential confounding variables, including neurological conditions (such as autonomic dysfunction and delirium), the existence of diabetes, and the influence of drugs (such as midazolam and opioids) that may impact PLR [46]. Despite these limitations, this study demonstrated the efficacy of an automated pupillometer as a predictive tool for early detection of brain herniation in acute malignant ischemic stroke patients. Notably, this study confirmed that other pupillary parameters (i.e., Size-initial, DV, and CH) in addition to the NPi score could be potential biomarkers for predicting brain herniation. Both groups will have an enhanced sample size, and other types of stroke patients will be included in future research. Moreover, data with a larger sample and multiple segmented time windows will be re-analyzed because our findings support that PLR variables 3 hours before the diagnosis of brain herniation were more determinant than PLR variables 6 hours before the diagnosis of brain herniation (see **Fig 3** vs. **S1 Fig**). Additionally, machine learning will be utilized to identify the most significant pupillary factors, contributing to improvements in the identification and prediction rate of brain herniation.

## 5. Conclusion

To our knowledge, this is the first study to investigate the quantitative characteristics of PLR as a biomarker for identifying and predicting brain herniation in acute stroke patients admitted to the Neuro-ICU. The findings of this study suggest that examining pupillary variables allows for timely triage of brain herniation. Additionally, changes in pupillary variables on the ipsilateral side are more significant than those on the contralateral side. These findings have practical importance since automated pupillometry can be a rapid, non-invasive, objective, and cost-effective technique for identifying brain herniation. Notably, automated pupillometry can provide early indications of brain herniation, enabling immediate and objective measurement, continuous monitoring, and timely interventions, improving patient outcomes while reducing the reliance on resource-intensive clinical assessments and brain imaging.

## Supporting information

S1 Table Statistical analysis results of eight pupillary variables as a function of the uniformly distributed (every 6-hour: 36-to-30, 30-to-24, 24-to-18, 18-to-12, 12-to-6, and 6-to-0 hours before the identification of the brain herniation) measurement timeframe on the ipsilateral and contralateral side for the herniation group. Values are presented as mean ± standard error. P indicates a *p* value.
(DOCX)

S2 Table Statistical analysis results of eight pupillary variables as a function of the measurement timeframe on the ipsilateral and contralateral sides for the herniation group. Values are presented as mean ± standard error. P indicates a *p* value.
(DOCX)

S3 Table Statistical analysis results of eight pupillary variables as a function of the measurement timeframe on the ipsilateral and contralateral side for the non-herniation group. Values are presented as mean ± standard error. P indicates a *p* value.
(DOCX)

S1 Fig Comparing features for pupil response on the ipsilateral (stroke-affected) and contralateral sides across the uniformly distributed past 36 hours (36-to-30, 30-to-24, 24-to-18, 18-to-12, 12-to-6, and 6-to-0 hours before the identification of the brain herniation) in the herniation group. (a) NPi; (b) Size-initial; (c) Size-min; (d) CH; (e) CV; (f) MCV; (g) CLAT; and (h) DV. Error bars indicate the standard error of the corresponding mean.
(TIFF)

## Abbreviations

APRoN: Automated pupillary response on Neuro-ICU
BMI: body mass index
MCA: Middle cerebral artery
ICA: Internal carotid artery
NIHSS: National institutes of health stroke scale
IQR: interquatile range
tPA: tissue plasmin activator
TTM: Targeted temperature management
mRS: Modified Rankin Scale
CAD: Coronary artery disease
TIA: Transient ischemic attack
HbA1c: Hemoglobin A1c
