# Supplementary material for: Automated quantitative pupillometry as a predictor for transtentorial brain herniation in patients with malignant acute ischemic stroke
Source: PLoS One. 2025 Jan 10;20(1):e0316358. doi: 10.1371/journal.pone.0316358 (PMC11723594; doi:10.1371/journal.pone.0316358)
Supplement: S1 Table — Values are presented as mean ± standard error. P indicates a p value. (DOCX) [file pone.0316358.s001.docx]

| Ipsilateral side | | | | | | | | | | | | | | | | | |
| --- | --- | --- | --- | --- | --- | --- | --- | --- | --- | --- | --- | --- | --- | --- | --- | --- | --- |
|  | Time | NPi | P | Size-initial | P | Size-min | P | CH | P | CV | P | MCV | P | CLAT | P | DV | P |
| ref | 36-to-30 | 4.51±0.07 | ­- | 2.78±0.28 | ­- | 2.07±0.13 | ­- | 23.36±3.87 | ­- | 1.51±0.31 | ­- | 2.12±0.46 | ­- | 0.25±0.01 | ­- | 0.54±0.12 | ­- |
|  | 30-to-24 | 4.58±0.07 | .367 | 2.98±0.22 | .310 | 2.11±0.12 | .751 | 27.56±2.65 | .035 | 1.67±0.20 | .412 | 2.52±0.28 | .200 | 0.26±0.01 | .576 | 0.72±0.10 | .071 |
|  | 24-to-18 | 3.98±0.32 | .110 | 2.51±0.21 | .148 | 2.14±0.24 | .660 | 15.25±2.57 | .091 | 0.76±0.14 | .032 | 1.21±0.22 | .076 | 0.27±0.01 | .203 | 0.34±0.06 | .153 |
|  | 18-to-12 | 3.92±0.44 | .190 | 2.67±0.40 | .668 | 2.30±0.41 | .469 | 14.57±2.75 | .067 | 0.83±0.18 | .065 | 1.27±0.27 | .115 | 0.27±0.02 | .505 | 0.33±0.08 | .206 |
|  | 12-to-6 | 3.69±0.35 | .016 | 3.18±0.29 | .165 | 2.67±0.27 | .004 | 15.45±2.62 | .083 | 1.02±0.22 | .214 | 1.51±0.28 | .273 | 0.28±0.01 | .031 | 0.48±0.09 | .724 |
|  | 6-to-0 | 2.98±0.38 | .000 | 3.72±0.34 | .001 | 3.23±0.29 | .000 | 12.73±3.48 | .029 | 1.00±0.39 | .298 | 1.58±0.55 | .419 | 0.31±0.03 | .034 | 0.37±0.10 | .289 |
| ref | 30-to-24 | 4.58±0.07 | ­- | 2.98±0.22 | ­- | 2.11±0.12 | ­- | 27.56±2.65 | ­- | 1.67±0.20 | - | 2.52±0.28 | - | 0.26±0.01 | - | 0.72±0.10 | ­- |
|  | 24-to-18 | 3.98±0.32 | .075 | 2.51±0.21 | .001 | 2.14±0.24 | .862 | 15.25±2.57 | .002 | 0.76±0.14 | .000 | 1.21±0.22 | .000 | 0.27±0.01 | .021 | 0.34±0.06 | .000 |
|  | 18-to-12 | 3.92±0.44 | .157 | 2.67±0.40 | .339 | 2.30±0.41 | .610 | 14.57±2.75 | .001 | 0.83±0.18 | .000 | 1.27±0.27 | .000 | 0.27±0.02 | .576 | 0.33±0.08 | .001 |
|  | 12-to-6 | 3.69±0.35 | .014 | 3.18±0.29 | .389 | 2.67±0.27 | .012 | 15.45±2.62 | .001 | 1.02±0.22 | .008 | 1.51±0.28 | .005 | 0.28±0.01 | .001 | 0.48±0.09 | .058 |
|  | 6-to-0 | 2.98±0.38 | .000 | 3.72±0.34 | .012 | 3.23±0.29 | .000 | 12.73±3.48 | .000 | 1.00±0.39 | .042 | 1.58±0.55 | .047 | 0.31±0.03 | .014 | 0.37±0.10 | .001 |
| ref | 24-to-18 | 3.98±0.32 | ­- | 2.51±0.21 | ­- | 2.14±0.24 | ­- | 15.25±2.57 | ­- | 0.76±0.14 | - | 1.21±0.22 | - | 0.27±0.01 | - | 0.34±0.06 | ­- |
|  | 18-to-12 | 3.92±0.44 | .835 | 2.67±0.40 | .574 | 2.30±0.41 | .554 | 14.57±2.75 | .734 | 0.83±0.18 | .506 | 1.27±0.27 | .670 | 0.27±0.02 | .712 | 0.33±0.08 | .742 |
|  | 12-to-6 | 3.69±0.35 | .227 | 3.18±0.29 | .003 | 2.67±0.27 | .009 | 15.45±2.62 | .914 | 1.02±0.22 | .085 | 1.51±0.28 | .120 | 0.28±0.01 | .320 | 0.48±0.09 | .043 |
|  | 6-to-0 | 2.98±0.38 | .001 | 3.72±0.34 | .000 | 3.23±0.29 | .000 | 12.73±3.48 | .437 | 1.00±0.39 | .435 | 1.58±0.55 | .365 | 0.31±0.03 | .066 | 0.37±0.10 | .657 |
| ref | 18-to-12 | 3.92±0.44 | ­- | 2.67±0.40 | ­- | 2.30±0.41 | ­- | 14.57±2.75 | ­- | 0.83±0.18 | - | 1.27±0.27 | - | 0.27±0.02 | - | 0.33±0.08 | ­- |
|  | 12-to-6 | 3.69±0.35 | .151 | 3.18±0.29 | .060 | 2.67±0.27 | .091 | 15.45±2.62 | .560 | 1.02±0.22 | .185 | 1.51±0.28 | .251 | 0.28±0.01 | .401 | 0.48±0.09 | .086 |
|  | 6-to-0 | 2.98±0.38 | .000 | 3.72±0.34 | .001 | 3.23±0.29 | .000 | 12.73±3.48 | .375 | 1.00±0.39 | .468 | 1.58±0.55 | .337 | 0.31±0.03 | .080 | 0.37±0.10 | .447 |
| ref | 12-to-6 | 3.69±0.35 | ­- | 3.18±0.29 | ­- | 2.67±0.27 | ­- | 15.45±2.62 | ­- | 1.02±0.22 | - | 1.51±0.28 | - | 0.28±0.01 | - | 0.48±0.09 | ­- |
|  | 6-to-0 | 2.98±0.38 | .007 | 3.72±0.34 | .021 | 3.23±0.29 | .002 | 12.73±3.48 | .222 | 1.00±0.39 | .939 | 1.58±0.55 | .859 | 0.31±0.03 | .099 | 0.37±0.10 | .206 |
| Contralateral side | | | | | | | | | | | | | | | | | |
|  | Time | NPi | P | Size-initial | P | Size-min | P | CH | P | CV | P | MCV | P | CLAT | P | DV | P |
| ref | 36-to-30 | 4.19±0.18 | - | 2.94±0.43 | - | 2.31±0.29 | - | 20.04±2.51 | - | 1.36±0.31 | - | 1.97±0.38 | - | 0.29±0.02 | - | 0.49±0.10 | - |
|  | 30-to-24 | 4.37±0.14 | .314 | 2.95±0.32 | .964 | 2.17±0.21 | .482 | 25.24±3.56 | .011 | 1.52±0.21 | .451 | 2.33±0.32 | .162 | 0.28±0.01 | .371 | 0.64±0.12 | .001 |
|  | 24-to-18 | 4.26±0.12 | .662 | 2.31±0.17 | .101 | 1.93±0.12 | .103 | 15.71±2.54 | .152 | 0.88±0.15 | .155 | 1.24±0.20 | .094 | 0.32±0.02 | .121 | 0.37±0.07 | .166 |
|  | 18-to-12 | 4.21±0.14 | .869 | 2.24±0.17 | .034 | 1.92±0.15 | .069 | 13.57±2.00 | .033 | 0.79±0.12 | .049 | 1.14±0.16 | .018 | 0.30±0.02 | .877 | 0.30±0.06 | .061 |
|  | 12-to-6 | 3.98±0.20 | .372 | 2.66±0.25 | .544 | 2.23±0.20 | .790 | 15.46±2.68 | .225 | 0.99±0.17 | .318 | 1.41±0.23 | .248 | 0.28±0.02 | .468 | 0.43±0.07 | .654 |
|  | 6-to-0 | 3.91±0.21 | .167 | 2.94±0.34 | .991 | 2.37±0.23 | .792 | 16.87±3.40 | .361 | 1.06±0.24 | .352 | 1.61±0.35 | .396 | 0.29±0.02 | .944 | 0.45±0.10 | .783 |
| ref | 30-to-24 | 4.37±0.14 | - | 2.95±0.32 | - | 2.17±0.21 | - | 25.24±3.56 | - | 1.52±0.21 | - | 2.33±0.32 | - | 0.28±0.01 | - | 0.64±0.12 | ­- |
|  | 24-to-18 | 4.26±0.12 | .151 | 2.31±0.17 | .006 | 1.93±0.12 | .077 | 15.71±2.54 | .001 | 0.88±0.15 | .001 | 1.24±0.20 | .000 | 0.32±0.02 | .037 | 0.37±0.07 | .009 |
|  | 18-to-12 | 4.21±0.14 | .249 | 2.24±0.17 | .002 | 1.92±0.15 | .056 | 13.57±2.00 | .001 | 0.79±0.12 | .000 | 1.14±0.16 | .000 | 0.30±0.02 | .312 | 0.30±0.06 | .001 |
|  | 12-to-6 | 3.98±0.20 | .018 | 2.66±0.25 | .339 | 2.23±0.20 | .688 | 15.46±2.68 | .014 | 0.99±0.17 | .047 | 1.41±0.23 | .021 | 0.28±0.02 | .906 | 0.43±0.07 | .099 |
|  | 6-to-0 | 3.91±0.21 | .029 | 2.94±0.34 | .958 | 2.37±0.23 | .313 | 16.87±3.40 | .014 | 1.06±0.24 | .030 | 1.61±0.35 | .022 | 0.29±0.02 | .292 | 0.45±0.10 | .096 |
| ref | 24-to-18 | 4.26±0.12 | - | 2.31±0.17 | - | 1.93±0.12 | - | 15.71±2.54 | - | 0.88±0.15 | - | 1.24±0.20 | - | 0.32±0.02 | - | 0.37±0.07 | ­- |
|  | 18-to-12 | 4.21±0.14 | .619 | 2.24±0.17 | .729 | 1.92±0.15 | .993 | 13.57±2.00 | .313 | 0.79±0.12 | .592 | 1.14±0.16 | .667 | 0.30±0.02 | .247 | 0.30±0.06 | .377 |
|  | 12-to-6 | 3.98±0.20 | .009 | 2.66±0.25 | .194 | 2.23±0.20 | .093 | 15.46±2.68 | .914 | 0.99±0.17 | .526 | 1.41±0.23 | .423 | 0.28±0.02 | .071 | 0.43±0.07 | .436 |
|  | 6-to-0 | 3.91±0.21 | .073 | 2.94±0.34 | .086 | 2.37±0.23 | .047 | 16.87±3.40 | .727 | 1.06±0.24 | .460 | 1.61±0.35 | .274 | 0.29±0.02 | .032 | 0.45±0.10 | .451 |
| ref | 18-to-12 | 4.21±0.14 | - | 2.24±0.17 | - | 1.92±0.15 | - | 13.57±2.00 | - | 0.79±0.12 | - | 1.14±0.16 | - | 0.30±0.02 | - | 0.30±0.06 | ­- |
|  | 12-to-6 | 3.98±0.20 | .120 | 2.66±0.25 | .080 | 2.23±0.20 | .071 | 15.46±2.68 | .430 | 0.99±0.17 | .224 | 1.41±0.23 | .270 | 0.28±0.02 | .445 | 0.43±0.07 | .057 |
|  | 6-to-0 | 3.91±0.21 | .044 | 2.94±0.34 | .002 | 2.37±0.23 | .004 | 16.87±3.40 | .237 | 1.06±0.24 | .053 | 1.61±0.35 | .044 | 0.29±0.02 | .933 | 0.45±0.10 | .014 |
| ref | 12-to-6 | 3.98±0.20 | - | 2.66±0.25 | - | 2.23±0.20 | - | 15.46±2.68 | - | 0.99±0.17 | - | 1.41±0.23 | - | 0.28±0.02 | - | 0.43±0.07 | ­- |
|  | 6-to-0 | 3.91±0.21 | .786 | 2.94±0.34 | .331 | 2.37±0.23 | .408 | 16.87±3.40 | .607 | 1.06±0.24 | .738 | 1.61±0.35 | .514 | 0.29±0.02 | .307 | 0.45±0.10 | .773 |
