# Supplementary material for: Automated quantitative pupillometry as a predictor for transtentorial brain herniation in patients with malignant acute ischemic stroke
Source: PLoS One. 2025 Jan 10;20(1):e0316358. doi: 10.1371/journal.pone.0316358 (PMC11723594; doi:10.1371/journal.pone.0316358)
Supplement: S2 Table — Values are presented as mean ± standard error. P indicates a p value. (DOCX) [file pone.0316358.s002.docx]

| psilateral side | | | | | | | | | | | | | | | | | |
| --- | --- | --- | --- | --- | --- | --- | --- | --- | --- | --- | --- | --- | --- | --- | --- | --- | --- |
|  | Time | NPi | P | Size-initial | P | Size-min | P | CH | P | CV | P | MCV | P | CLAT | P | DV | P |
| ref | 48-to-33 | 4.47±0.15 | ­- | 2.68±0.12 | ­- | 2.04±0.10 | ­- | 22.63±2.91 | ­- | 1.38±0.18 | ­- | 1.97±0.23 | ­- | 0.25±0.01 | ­- | 0.57±0.05 | ­- |
|  | 33-to-27 | 4.55±0.05 | .517 | 2.94±0.27 | .156 | 2.13±0.12 | .323 | 25.57±3.45 | .382 | 1.68±0.24 | .013 | 2.34±0.37 | .074 | 0.26±0.01 | .235 | 0.60±0.11 | .800 |
|  | 27-to-21 | 4.26±0.21 | .348 | 2.61±0.18 | .594 | 2.11±0.17 | .544 | 18.57±2.43 | .200 | 1.01±0.14 | .155 | 1.61±0.22 | .320 | 0.28±0.02 | .062 | 0.49±0.08 | .349 |
|  | 21-to-15 | 3.82±0.43 | .140 | 2.51±0.30 | .490 | 2.21±0.32 | .549 | 13.57±2.29 | .010 | 0.66±0.12 | .002 | 1.01±0.15 | .000 | 0.26±0.02 | .406 | 0.28±0.06 | .000 |
|  | 15-to-9 | 3.63±0.50 | .049 | 2.97±0.42 | .423 | 2.55±0.42 | .145 | 14.41±3.30 | .020 | 0.87±0.24 | .136 | 1.38±0.32 | .159 | 0.27±0.02 | .178 | 0.41±0.08 | .076 |
|  | 9-to-3 | 3.57±0.33 | .009 | 3.24±0.29 | .033 | 2.72±0.22 | .002 | 14.72±3.19 | .022 | 1.09±0.33 | .420 | 1.59±0.45 | .395 | 0.28±0.01 | .064 | 0.44±0.09 | .099 |
|  | 3-to-0 | 1.80±0.44 | .000 | 4.22±0.41 | .000 | 4.01±0.40 | .000 | 5.31±0.82 | .000 | 0.35±0.07 | .000 | 0.75±0.07 | .000 | 0.38±0.05 | .015 | 0.18±0.03 | .000 |
| ref | 33-to-27 | 4.55±0.05 | ­- | 2.94±0.27 | ­- | 2.13±0.12 | ­- | 25.57±3.45 | ­- | 1.68±0.24 | ­- | 2.34±0.37 | ­- | 0.26±0.01 | ­- | 0.60±0.11 | ­- |
|  | 27-to-21 | 4.26±0.21 | .190 | 2.61±0.18 | .169 | 2.11±0.17 | .894 | 18.57±2.43 | .116 | 1.01±0.14 | .029 | 1.61±0.22 | .128 | 0.28±0.02 | .058 | 0.49±0.08 | .472 |
|  | 21-to-15 | 3.82±0.43 | .087 | 2.51±0.30 | .106 | 2.21±0.32 | .771 | 13.57±2.29 | .007 | 0.66±0.12 | .000 | 1.01±0.15 | .001 | 0.26±0.02 | .607 | 0.28±0.06 | .017 |
|  | 15-to-9 | 3.63±0.50 | .061 | 2.97±0.42 | .948 | 2.55±0.42 | .271 | 14.41±3.30 | .032 | 0.87±0.24 | .034 | 1.38±0.32 | .076 | 0.27±0.02 | .269 | 0.41±0.08 | .199 |
|  | 9-to-3 | 3.57±0.33 | .003 | 3.24±0.29 | .402 | 2.72±0.22 | .008 | 14.72±3.19 | .022 | 1.09±0.33 | .142 | 1.59±0.45 | .187 | 0.28±0.01 | .113 | 0.44±0.09 | .224 |
|  | 3-to-0 | 1.80±0.44 | .000 | 4.22±0.41 | .002 | 4.01±0.40 | .000 | 5.31±0.82 | .000 | 0.35±0.07 | .000 | 0.75±0.07 | .000 | 0.38±0.05 | .017 | 0.18±0.03 | .000 |
| ref | 27-to-21 | 4.26±0.21 | ­- | 2.61±0.18 | ­- | 2.11±0.17 | ­- | 18.57±2.43 | ­- | 1.01±0.14 | ­- | 1.61±0.22 | ­- | 0.28±0.02 | ­- | 0.49±0.08 | ­- |
|  | 21-to-15 | 3.82±0.43 | .122 | 2.51±0.30 | .618 | 2.21±0.32 | .595 | 13.57±2.29 | .030 | 0.66±0.12 | .005 | 1.01±0.15 | .002 | 0.26±0.02 | .383 | 0.28±0.06 | .009 |
|  | 15-to-9 | 3.63±0.50 | .044 | 2.97±0.42 | .284 | 2.55±0.42 | .137 | 14.41±3.30 | .079 | 0.87±0.24 | .353 | 1.38±0.32 | .276 | 0.27±0.02 | .611 | 0.41±0.08 | .244 |
|  | 9-to-3 | 3.57±0.33 | .000 | 3.24±0.29 | .017 | 2.72±0.22 | .000 | 14.72±3.19 | .087 | 1.09±0.33 | .744 | 1.59±0.45 | .933 | 0.28±0.01 | .650 | 0.44±0.09 | .517 |
|  | 3-to-0 | 1.80±0.44 | .000 | 4.22±0.41 | .000 | 4.01±0.40 | .000 | 5.31±0.82 | .000 | 0.35±0.07 | .000 | 0.75±0.07 | .000 | 0.38±0.05 | .052 | 0.18±0.03 | .000 |
| ref | 21-to-15 | 3.82±0.43 | ­- | 2.51±0.30 | ­- | 2.21±0.32 | ­- | 13.57±2.29 | ­- | 0.66±0.12 | ­- | 1.01±0.15 | ­- | 0.26±0.02 | ­- | 0.28±0.06 | ­- |
|  | 15-to-9 | 3.63±0.50 | .546 | 2.97±0.42 | .125 | 2.55±0.42 | .209 | 14.41±3.30 | .676 | 0.87±0.24 | .170 | 1.38±0.32 | .106 | 0.27±0.02 | .701 | 0.41±0.08 | .030 |
|  | 9-to-3 | 3.57±0.33 | .373 | 3.24±0.29 | .011 | 2.72±0.22 | .023 | 14.72±3.19 | .658 | 1.09±0.33 | .086 | 1.59±0.45 | .105 | 0.28±0.01 | .277 | 0.44±0.09 | .031 |
|  | 3-to-0 | 1.80±0.44 | .000 | 4.22±0.41 | .000 | 4.01±0.40 | .000 | 5.31±0.82 | .000 | 0.35±0.07 | .037 | 0.75±0.07 | .135 | 0.38±0.05 | .011 | 0.18±0.03 | .139 |
| ref | 15-to-9 | 3.63±0.50 | ­- | 2.97±0.42 | ­- | 2.55±0.42 | ­- | 14.41±3.30 | ­- | 0.87±0.24 | ­- | 1.38±0.32 | ­- | 0.27±0.02 | ­- | 0.41±0.08 | ­- |
|  | 9-to-3 | 3.57±0.33 | .853 | 3.24±0.29 | .464 | 2.72±0.22 | .594 | 14.72±3.19 | .846 | 1.09±0.33 | .274 | 1.59±0.45 | .388 | 0.28±0.01 | .951 | 0.44±0.09 | .662 |
|  | 3-to-0 | 1.80±0.44 | .000 | 4.22±0.41 | .000 | 4.01±0.40 | .000 | 5.31±0.82 | .006 | 0.35±0.07 | .035 | 0.75±0.07 | .057 | 0.38±0.05 | .071 | 0.18±0.03 | .006 |
| ref | 9-to-3 | 3.57±0.33 | ­- | 3.24±0.29 | ­- | 2.72±0.22 | ­- | 14.72±3.19 | ­- | 1.09±0.33 | ­- | 1.59±0.45 | ­- | 0.28±0.01 | ­- | 0.44±0.09 | ­- |
|  | 3-to-0 | 1.80±0.44 | .000 | 4.22±0.41 | .001 | 4.01±0.40 | .000 | 5.31±0.82 | .003 | 0.35±0.07 | .020 | 0.75±0.07 | .055 | 0.38±0.05 | .022 | 0.18±0.03 | .002 |
| **Contralateral side** | | | | | | | | | | | | | | | | | |
|  | Time | NPi | P | Size-initial | P | Size-min | P | CH | P | CV | P | MCV | P | CLAT | P | DV | P |
| ref | 48-to-33 | 4.13±0.23 | - | 2.68±0.23 | ­- | 2.21±0.22 | ­- | 17.41±2.16 | ­- | 1.10±0.08 | ­- | 1.66±0.10 | ­- | 0.30±0.02 | ­- | 0.48±0.08 | ­- |
|  | 33-to-27 | 4.24±0.12 | .535 | 3.05±0.34 | .013 | 2.33±0.23 | .041 | 22.39±2.71 | .178 | 1.53±0.24 | .038 | 2.27±0.33 | .081 | 0.29±0.02 | .641 | 0.54±0.10 | .545 |
|  | 27-to-21 | 4.37±0.13 | .207 | 2.51±0.26 | .451 | 1.98±0.17 | .184 | 19.33±2.83 | .484 | 1.11±0.18 | .960 | 1.59±0.25 | .739 | 0.29±0.02 | .832 | 0.47±0.09 | .936 |
|  | 21-to-15 | 4.22±0.12 | .654 | 2.18±0.16 | .001 | 1.87±0.12 | .025 | 13.76±2.11 | .098 | 0.76±0.13 | .001 | 1.06±0.17 | .002 | 0.31±0.02 | .508 | 0.27±0.06 | .001 |
|  | 15-to-9 | 3.78±0.40 | .284 | 2.48±0.30 | .518 | 2.18±0.30 | .921 | 12.53±2.52 | .034 | 0.83±0.16 | .115 | 1.15±0.17 | .038 | 0.31±0.03 | .663 | 0.39±0.08 | .421 |
|  | 9-to-3 | 4.16±0.16 | .915 | 2.59±0.24 | .794 | 2.12±0.18 | .746 | 16.57±2.96 | .774 | 1.01±0.19 | .680 | 1.50±0.28 | .622 | 0.29±0.02 | .709 | 0.43±0.08 | .666 |
|  | 3-to-0 | 3.47±0.32 | 0.100 | 2.95±0.49 | .572 | 2.54±0.34 | .335 | 11.28±3.67 | .225 | 0.69±0.28 | .130 | 1.06±0.40 | .189 | 0.30±0.02 | .974 | 0.29±0.12 | .188 |
| ref | 33-to-27 | 4.24±0.12 | ­- | 3.05±0.34 | ­- | 2.33±0.23 | ­- | 22.39±2.71 | ­- | 1.53±0.24 | ­- | 2.27±0.33 | ­- | 0.29±0.02 | ­- | 0.54±0.10 | ­- |
|  | 27-to-21 | 4.37±0.13 | .267 | 2.51±0.26 | .087 | 1.98±0.17 | .073 | 19.33±2.83 | .234 | 1.11±0.18 | .092 | 1.59±0.25 | .056 | 0.29±0.02 | .939 | 0.47±0.09 | .456 |
|  | 21-to-15 | 4.22±0.12 | .858 | 2.18±0.16 | .001 | 1.87±0.12 | .007 | 13.76±2.11 | .002 | 0.76±0.13 | .001 | 1.06±0.17 | .000 | 0.31±0.02 | .246 | 0.27±0.06 | .002 |
|  | 15-to-9 | 3.78±0.40 | .254 | 2.48±0.30 | .112 | 2.18±0.30 | .600 | 12.53±2.52 | .003 | 0.83±0.16 | .007 | 1.15±0.17 | .001 | 0.31±0.03 | .515 | 0.39±0.08 | .147 |
|  | 9-to-3 | 4.16±0.16 | .610 | 2.59±0.24 | .296 | 2.12±0.18 | .433 | 16.57±2.96 | .104 | 1.01±0.19 | .128 | 1.50±0.28 | .109 | 0.29±0.02 | .882 | 0.43±0.08 | .425 |
|  | 3-to-0 | 3.47±0.32 | .019 | 2.95±0.49 | .832 | 2.54±0.34 | .523 | 11.28±3.67 | .004 | 0.69±0.28 | .002 | 1.06±0.40 | .001 | 0.30±0.02 | .912 | 0.29±0.12 | .035 |
| ref | 27-to-21 | 4.37±0.13 | ­- | 2.51±0.26 | ­- | 1.98±0.17 | ­- | 19.33±2.83 | ­- | 1.11±0.18 | ­- | 1.59±0.25 | ­- | 0.29±0.02 | ­- | 0.47±0.09 | ­- |
|  | 21-to-15 | 4.22±0.12 | .104 | 2.18±0.16 | .088 | 1.87±0.12 | .353 | 13.76±2.11 | .010 | 0.76±0.13 | .012 | 1.06±0.17 | .014 | 0.31±0.02 | .336 | 0.27±0.06 | .001 |
|  | 15-to-9 | 3.78±0.40 | .060 | 2.48±0.30 | .920 | 2.18±0.30 | .308 | 12.53±2.52 | .047 | 0.83±0.16 | .269 | 1.15±0.17 | .161 | 0.31±0.03 | .458 | 0.39±0.08 | .454 |
|  | 9-to-3 | 4.16±0.16 | .254 | 2.59±0.24 | .801 | 2.12±0.18 | .502 | 16.57±2.96 | .378 | 1.01±0.19 | .603 | 1.50±0.28 | .778 | 0.29±0.02 | .584 | 0.43±0.08 | .679 |
|  | 3-to-0 | 3.47±0.32 | .004 | 2.95±0.49 | .433 | 2.54±0.34 | .109 | 11.28±3.67 | .123 | 0.69±0.28 | .237 | 1.06±0.40 | .315 | 0.30±0.02 | .924 | 0.29±0.12 | .272 |
| ref | 21-to-15 | 4.22±0.12 | ­- | 2.18±0.16 | ­- | 1.87±0.12 | ­- | 13.76±2.11 | ­- | 0.76±0.13 | ­- | 1.06±0.17 | ­- | 0.31±0.02 | ­- | 0.27±0.06 | ­- |
|  | 15-to-9 | 3.78±0.40 | .201 | 2.48±0.30 | .314 | 2.18±0.30 | .233 | 12.53±2.52 | .593 | 0.83±0.16 | .688 | 1.15±0.17 | .654 | 0.31±0.03 | .915 | 0.39±0.08 | .248 |
|  | 9-to-3 | 4.16±0.16 | .671 | 2.59±0.24 | .146 | 2.12±0.18 | .168 | 16.57±2.96 | .356 | 1.01±0.19 | .219 | 1.50±0.28 | .140 | 0.29±0.02 | .236 | 0.43±0.08 | .123 |
|  | 3-to-0 | 3.47±0.32 | .003 | 2.95±0.49 | .097 | 2.54±0.34 | .023 | 11.28±3.67 | .524 | 0.69±0.28 | .798 | 1.06±0.40 | .994 | 0.30±0.02 | .661 | 0.29±0.12 | .917 |
| ref | 15-to-9 | 3.78±0.40 | ­- | 2.48±0.30 | ­- | 2.18±0.30 | ­- | 12.53±2.52 | ­- | 0.83±0.16 | ­- | 1.15±0.17 | ­- | 0.31±0.03 | ­- | 0.39±0.08 | ­- |
|  | 9-to-3 | 4.16±0.16 | .371 | 2.59±0.24 | .737 | 2.12±0.18 | .837 | 16.57±2.96 | .140 | 1.01±0.19 | .346 | 1.50±0.28 | .125 | 0.29±0.02 | .318 | 0.43±0.08 | .570 |
|  | 3-to-0 | 3.47±0.32 | .513 | 2.95±0.49 | .254 | 2.54±0.34 | .281 | 11.28±3.67 | .747 | 0.69±0.28 | .477 | 1.06±0.40 | .775 | 0.30±0.02 | .635 | 0.29±0.12 | .278 |
| ref | 9-to-3 | 4.16±0.16 | ­- | 2.59±0.24 | ­- | 2.12±0.18 | ­- | 16.57±2.96 | ­- | 1.01±0.19 | ­- | 1.50±0.28 | ­- | 0.29±0.02 | ­- | 0.43±0.08 | ­- |
|  | 3-to-0 | 3.47±0.32 | .013 | 2.95±0.49 | .463 | 2.54±0.34 | .144 | 11.28±3.67 | .239 | 0.69±0.28 | .355 | 1.06±0.40 | .369 | 0.30±0.02 | .792 | 0.29±0.12 | .335 |
