# Supplementary material for: Automated quantitative pupillometry as a predictor for transtentorial brain herniation in patients with malignant acute ischemic stroke
Source: PLoS One. 2025 Jan 10;20(1):e0316358. doi: 10.1371/journal.pone.0316358 (PMC11723594; doi:10.1371/journal.pone.0316358)
Supplement: S3 Table — Values are presented as mean ± standard error. P indicates a p value. (DOCX) [file pone.0316358.s003.docx]

| Ipsilateral side | | | | | | | | | | | | | | | | | |
| --- | --- | --- | --- | --- | --- | --- | --- | --- | --- | --- | --- | --- | --- | --- | --- | --- | --- |
|  | Time | NPi | P | Size-initial | P | Size-min | P | CH | P | CV | P | MCV | P | CLAT | P | DV | P |
| ref | 48-to-33 | 4.08±0.11 | ­- | 3.99±0.12 | ­- | 2.81±0.09 | ­- | 29.05±1.31 | ­- | 2.09±0.12 | ­- | 3.27±0.18 | ­- | 0.26±0.01 | ­- | 0.94±0.05 | ­- |
|  | 33-to-27 | 4.06±0.11 | .779 | 3.98±0.12 | .896 | 2.79±0.10 | .894 | 29.02±1.40 | .890 | 2.12±0.13 | .747 | 3.29±0.18 | .870 | 0.26±0.01 | .752 | 0.88±0.05 | .229 |
|  | 27-to-21 | 4.01±0.13 | .285 | 4.00±0.14 | .907 | 2.85±0.11 | .516 | 28.23±1.42 | .395 | 2.07±0.12 | .804 | 3.08±0.18 | .168 | 0.25±0.01 | .505 | 0.86±0.05 | .040 |
|  | 21-to-15 | 4.05±0.11 | .700 | 4.01±0.12 | .859 | 2.83±0.09 | .683 | 28.76±1.35 | .749 | 2.09±0.12 | .949 | 3.27±0.18 | .979 | 0.26±0.01 | .478 | 0.93±0.05 | .930 |
|  | 15-to-9 | 3.97±0.12 | .122 | 4.11±0.12 | .363 | 2.90±0.10 | .234 | 28.96±1.49 | .927 | 2.06±0.13 | .733 | 3.27±0.20 | .984 | 0.26±0.01 | .522 | 0.93±0.05 | .790 |
|  | 9-to-3 | 4.13±0.11 | .637 | 3.91±0.12 | .424 | 2.75±0.09 | .502 | 29.02±1.25 | .977 | 2.02±0.12 | .390 | 3.18±0.17 | .433 | 0.26±0.01 | .300 | 0.93±0.04 | .924 |
|  | 3-to-0 | 3.97±0.13 | .203 | 4.04±0.12 | .656 | 2.90±0.10 | .260 | 28.04±1.38 | .364 | 2.06±0.13 | .766 | 3.35±0.21 | .637 | 0.27±0.01 | .144 | 0.96±0.05 | .567 |
| ref | 33-to-27 | 4.06±0.11 | ­- | 3.98±0.12 | ­- | 2.79±0.10 | ­- | 29.02±1.40 | ­- | 2.12±0.13 | - | 3.29±0.18 | - | 0.26±0.01 | - | 0.88±0.05 | ­- |
|  | 27-to-21 | 4.01±0.13 | .435 | 4.00±0.14 | .811 | 2.85±0.11 | .444 | 28.23±1.42 | .375 | 2.07±0.12 | .655 | 3.08±0.18 | .196 | 0.25±0.01 | .440 | 0.86±0.05 | .695 |
|  | 21-to-15 | 4.05±0.11 | .907 | 4.01±0.12 | .759 | 2.83±0.09 | .627 | 28.76±1.35 | .677 | 2.09±0.12 | .726 | 3.27±0.18 | .894 | 0.26±0.01 | .732 | 0.93±0.05 | .296 |
|  | 15-to-9 | 3.97±0.12 | .256 | 4.11±0.12 | .319 | 2.90±0.10 | .232 | 28.96±1.49 | .827 | 2.06±0.13 | .559 | 3.27±0.20 | .901 | 0.26±0.01 | .698 | 0.93±0.05 | .233 |
|  | 9-to-3 | 4.13±0.11 | .486 | 3.91±0.12 | .571 | 2.75±0.09 | .592 | 29.02±1.25 | .869 | 2.02±0.12 | .322 | 3.18±0.17 | .418 | 0.26±0.01 | .482 | 0.93±0.04 | .216 |
|  | 3-to-0 | 3.97±0.13 | .315 | 4.04±0.12 | .607 | 2.90±0.10 | .253 | 28.04±1.38 | .368 | 2.06±0.13 | .639 | 3.35±0.21 | .755 | 0.27±0.01 | .152 | 0.96±0.05 | .143 |
| ref | 27-to-21 | 4.01±0.13 | ­- | 4.00±0.14 | ­- | 2.85±0.11 | ­- | 28.23±1.42 | ­- | 2.07±0.12 | - | 3.08±0.18 | - | 0.25±0.01 | - | 0.86±0.05 | ­- |
|  | 21-to-15 | 4.05±0.11 | .665 | 4.01±0.12 | .974 | 2.83±0.09 | .789 | 28.76±1.35 | .628 | 2.09±0.12 | .884 | 3.27±0.18 | .222 | 0.26±0.01 | .277 | 0.93±0.05 | .124 |
|  | 15-to-9 | 3.97±0.12 | .729 | 4.11±0.12 | .471 | 2.90±0.10 | .594 | 28.96±1.49 | .547 | 2.06±0.13 | .946 | 3.27±0.20 | .254 | 0.26±0.01 | .240 | 0.93±0.05 | .083 |
|  | 9-to-3 | 4.13±0.11 | .233 | 3.91±0.12 | .378 | 2.75±0.09 | .210 | 29.02±1.25 | .482 | 2.02±0.12 | .623 | 3.18±0.17 | .457 | 0.26±0.01 | .149 | 0.93±0.04 | .073 |
|  | 3-to-0 | 3.97±0.13 | .718 | 4.04±0.12 | .742 | 2.90±0.10 | .588 | 28.04±1.38 | .878 | 2.06±0.13 | .938 | 3.35±0.21 | .110 | 0.27±0.01 | .150 | 0.96±0.05 | .032 |
| ref | 21-to-15 | 4.05±0.11 | ­- | 4.01±0.12 | ­- | 2.83±0.09 | ­- | 28.76±1.35 | ­- | 2.09±0.12 | - | 3.27±0.18 | - | 0.26±0.01 | - | 0.93±0.05 | ­- |
|  | 15-to-9 | 3.97±0.12 | .333 | 4.11±0.12 | .337 | 2.90±0.10 | .327 | 28.96±1.49 | .860 | 2.06±0.13 | .796 | 3.27±0.20 | .999 | 0.26±0.01 | .896 | 0.93±0.05 | .897 |
|  | 9-to-3 | 4.13±0.11 | .296 | 3.91±0.12 | .314 | 2.75±0.09 | .221 | 29.02±1.25 | .737 | 2.02±0.12 | .433 | 3.18±0.17 | .456 | 0.26±0.01 | .651 | 0.93±0.04 | .994 |
|  | 3-to-0 | 3.97±0.13 | .389 | 4.04±0.12 | .715 | 2.90±0.10 | .331 | 28.04±1.38 | .538 | 2.06±0.13 | .830 | 3.35±0.21 | .657 | 0.27±0.01 | .477 | 0.96±0.05 | .556 |
| ref | 15-to-9 | 3.97±0.12 | ­- | 4.11±0.12 | ­- | 2.90±0.10 | ­- | 28.96±1.49 | ­- | 2.06±0.13 | - | 3.27±0.20 | - | 0.26±0.01 | - | 0.93±0.05 | ­- |
|  | 9-to-3 | 4.13±0.11 | .069 | 3.91±0.12 | .112 | 2.75±0.09 | .073 | 29.02±1.25 | .943 | 2.02±0.12 | .664 | 3.18±0.17 | .483 | 0.26±0.01 | .792 | 0.93±0.04 | .837 |
|  | 3-to-0 | 3.97±0.13 | .969 | 4.04±0.12 | .626 | 2.90±0.10 | .982 | 28.04±1.38 | .468 | 2.06±0.13 | .987 | 3.35±0.21 | .706 | 0.27±0.01 | .587 | 0.96±0.05 | .360 |
| ref | 9-to-3 | 4.13±0.11 | ­- | 3.91±0.12 | ­- | 2.75±0.09 | ­- | 29.02±1.25 | ­- | 2.02±0.12 | - | 3.18±0.17 | - | 0.26±0.01 | - | 0.93±0.04 | ­- |
|  | 3-to-0 | 3.97±0.13 | .043 | 4.04±0.12 | .188 | 2.90±0.10 | .035 | 28.04±1.38 | .331 | 2.06±0.13 | .743 | 3.35±0.21 | .251 | 0.27±0.01 | .582 | 0.96±0.05 | .501 |
| Contralateral side | | | | | | | | | | | | | | | | | |
|  | Time | NPi | P | Size-initial | P | Size-min | P | CH | P | CV | P | MCV | P | CLAT | P | DV | P |
| ref | 48-to-33 | 4.00±0.12 | - | 3.98±0.12 | ­- | 2.85±0.09 | ­- | 27.96±1.20 | ­- | 2.12±0.13 | ­- | 3.21±0.18 | ­- | 0.26±0.01 | ­- | 0.94±0.05 | ­- |
|  | 33-to-27 | 4.11±0.12 | .076 | 3.92±0.12 | .534 | 2.75±0.10 | .158 | 29.36±1.41 | .131 | 2.12±0.12 | .977 | 3.24±0.18 | .803 | 0.26±0.01 | .800 | 0.93±0.05 | .647 |
|  | 27-to-21 | 4.01±0.13 | .895 | 4.05±0.15 | .481 | 2.86±0.11 | .909 | 28.87±1.42 | .374 | 2.21±0.14 | .306 | 3.28±0.19 | .579 | 0.26±0.01 | .714 | 0.88±0.05 | .141 |
|  | 21-to-15 | 4.03±0.11 | .654 | 4.02±0.11 | .663 | 2.86±0.09 | .845 | 28.16±1.22 | .809 | 2.12±0.13 | .986 | 3.23±0.18 | .834 | 0.26±0.01 | .437 | 0.95±0.04 | .790 |
|  | 15-to-9 | 4.08±0.11 | .406 | 4.02±0.14 | .752 | 2.80±0.10 | .567 | 29.53±1.55 | .150 | 2.12±0.14 | .995 | 3.32±0.23 | .460 | 0.26±0.01 | .753 | 0.97±0.06 | .633 |
|  | 9-to-3 | 4.05±0.11 | .661 | 3.88±0.12 | .308 | 2.76±0.09 | .319 | 28.09±1.20 | .883 | 2.01±0.12 | .229 | 3.08±0.16 | .305 | 0.27±0.01 | .039 | 0.93±0.05 | .762 |
|  | 3-to-0 | 3.96±0.12 | .688 | 4.02±0.13 | .753 | 2.89±0.10 | .625 | 27.56±1.30 | .711 | 2.07±0.13 | .611 | 3.15±0.18 | .724 | 0.27±0.01 | .351 | 0.96±0.05 | .682 |
| ref | 33-to-27 | 4.11±0.12 | - | 3.92±0.12 | - | 2.75±0.10 | - | 29.36±1.41 | - | 2.12±0.12 | - | 3.24±0.18 | - | 0.26±0.01 | - | 0.93±0.05 | - |
|  | 27-to-21 | 4.01±0.13 | .238 | 4.05±0.15 | .203 | 2.86±0.11 | .164 | 28.87±1.42 | .696 | 2.21±0.14 | .388 | 3.28±0.19 | .780 | 0.26±0.01 | .890 | 0.88±0.05 | .333 |
|  | 21-to-15 | 4.03±0.11 | .348 | 4.02±0.11 | .332 | 2.86±0.09 | .158 | 28.16±1.22 | .224 | 2.12±0.13 | .991 | 3.23±0.18 | .953 | 0.26±0.01 | .789 | 0.95±0.04 | .441 |
|  | 15-to-9 | 4.08±0.11 | .700 | 4.02±0.14 | .466 | 2.80±0.10 | .652 | 29.53±1.55 | .891 | 2.12±0.14 | .987 | 3.32±0.23 | .604 | 0.26±0.01 | .657 | 0.97±0.06 | .427 |
|  | 9-to-3 | 4.05±0.11 | .512 | 3.88±0.12 | .707 | 2.76±0.09 | .924 | 28.09±1.20 | .210 | 2.01±0.12 | .240 | 3.08±0.16 | .236 | 0.27±0.01 | .217 | 0.93±0.05 | .875 |
|  | 3-to-0 | 3.96±0.12 | .134 | 4.02±0.13 | .442 | 2.89±0.10 | .157 | 27.56±1.30 | .144 | 2.07±0.13 | .654 | 3.15±0.18 | .611 | 0.27±0.01 | .534 | 0.96±0.05 | .458 |
| ref | 27-to-21 | 4.01±0.13 | - | 4.05±0.15 | - | 2.86±0.11 | - | 28.87±1.42 | - | 2.21±0.14 | - | 3.28±0.19 | - | 0.26±0.01 | - | 0.88±0.05 | ­- |
|  | 21-to-15 | 4.03±0.11 | .822 | 4.02±0.11 | .737 | 2.86±0.09 | .972 | 28.16±1.22 | .546 | 2.12±0.13 | .354 | 3.23±0.18 | .738 | 0.26±0.01 | .870 | 0.95±0.04 | .097 |
|  | 15-to-9 | 4.08±0.11 | .555 | 4.02±0.14 | .802 | 2.80±0.10 | .550 | 29.53±1.55 | .581 | 2.12±0.14 | .370 | 3.32±0.23 | .805 | 0.26±0.01 | .550 | 0.97±0.06 | .115 |
|  | 9-to-3 | 4.05±0.11 | .787 | 3.88±0.12 | .115 | 2.76±0.09 | .336 | 28.09±1.20 | .500 | 2.01±0.12 | .030 | 3.08±0.16 | .146 | 0.27±0.01 | .207 | 0.93±0.05 | .258 |
|  | 3-to-0 | 3.96±0.12 | .686 | 4.02±0.13 | .772 | 2.89±0.10 | .739 | 27.56±1.30 | .267 | 2.07±0.13 | .190 | 3.15±0.18 | .425 | 0.27±0.01 | .584 | 0.96±0.05 | .087 |
| ref | 21-to-15 | 4.03±0.11 | - | 4.02±0.11 | - | 2.86±0.09 | - | 28.16±1.22 | - | 2.12±0.13 | - | 3.23±0.18 | - | 0.26±0.01 | - | 0.95±0.04 | ­- |
|  | 15-to-9 | 4.08±0.11 | .541 | 4.02±0.14 | .971 | 2.80±0.10 | .399 | 29.53±1.55 | .201 | 2.12±0.14 | .992 | 3.32±0.23 | .531 | 0.26±0.01 | .447 | 0.97±0.06 | .746 |
|  | 9-to-3 | 4.05±0.11 | .874 | 3.88±0.12 | .143 | 2.76±0.09 | .193 | 28.09±1.20 | .943 | 2.01±0.12 | .219 | 3.08±0.16 | .219 | 0.27±0.01 | .124 | 0.93±0.05 | .530 |
|  | 3-to-0 | 3.96±0.12 | .411 | 4.02±0.13 | .991 | 2.89±0.10 | .678 | 27.56±1.30 | .608 | 2.07±0.13 | .637 | 3.15±0.18 | .616 | 0.27±0.01 | .635 | 0.96±0.05 | .796 |
| ref | 15-to-9 | 4.08±0.11 | - | 4.02±0.14 | - | 2.80±0.10 | - | 29.53±1.55 | - | 2.12±0.14 | - | 3.32±0.23 | - | 0.26±0.01 | - | 0.97±0.06 | ­- |
|  | 9-to-3 | 4.05±0.11 | .638 | 3.88±0.12 | .219 | 2.76±0.09 | .671 | 28.09±1.20 | .162 | 2.01±0.12 | .273 | 3.08±0.16 | .122 | 0.27±0.01 | .065 | 0.93±0.05 | .450 |
|  | 3-to-0 | 3.96±0.12 | .144 | 4.02±0.13 | .983 | 2.89±0.10 | .299 | 27.56±1.30 | .106 | 2.07±0.13 | .604 | 3.15±0.18 | .359 | 0.27±0.01 | .301 | 0.96±0.05 | .919 |
| ref | 9-to-3 | 4.05±0.11 | - | 3.88±0.12 | - | 2.76±0.09 | - | 28.09±1.20 | - | 2.01±0.12 | - | 3.08±0.16 | - | 0.27±0.01 | - | 0.93±0.05 | ­- |
|  | 3-to-0 | 3.96±0.12 | .242 | 4.02±0.13 | .204 | 2.89±0.10 | .105 | 27.56±1.30 | .546 | 2.07±0.13 | .506 | 3.15±0.18 | .573 | 0.27±0.01 | .429 | 0.96±0.05 | .411 |
